# A Multimodal Analysis of Online Information Foraging in Health-Related Topics Based on Stimulus-Engagement Alignment: Observational Feasibility Study

**DOI:** 10.2196/64901

**Published:** 2025-07-14

**Authors:** Szilvia Zörgő, Gjalt-Jorn Peters, Anna Jeney, Szilárd Dávid Kovács, Rik Crutzen

**Affiliations:** 1 Faculty of Health, Medicine and Life Sciences, Maastricht University, P.O. Box 616, Maastricht, 6200 MD, The Netherlands, 31 308622466; 2 Faculty of Psychology, Open University of the Netherlands, Heerlen, The Netherlands; 3 College of Social Sciences, Seoul National University, Seoul, Republic of Korea; 4 Faculty of Medicine, Semmelweis University, Budapest, Hungary

**Keywords:** methodology, information appraisal, multimodal data, data visualization, digital health literacy, information-seeking, credibility, health information, information foraging, information retrieval

## Abstract

**Background:**

The recent increase in online health information–seeking has prompted extensive user appraisal of encountered content. Information consumption depends crucially on the quality of encountered information and the user’s ability to evaluate it; yet, within the context of web-based, organic search behavior, few studies take into account both these aspects simultaneously.

**Objective:**

We aimed to explore a method to bridge these two aspects and grant even consideration to both the stimulus (web page content) and the user (ability to appraise encountered content). We examined novices and experts in information retrieval and appraisal to demonstrate a novel approach to studying information foraging theory: stimulus-engagement alignment (SEA).

**Methods:**

We sampled from experts and novices in information retrieval and assessment, asking participants to conduct a 10-minute search task with a specific information goal. We used an observational and a retrospective think-aloud protocol to collect data within the framework of an interview. Data from 3 streams (think-aloud, human-computer interaction, and screen content) were manually coded in the Reproducible Open Coding Kit standard and subsequently aligned and represented in a tabularized format with the R package {rock}. SEA scores were derived from designated code co-occurrences in specific segments of data within the stimulus data stream versus the think-aloud and human-computer interaction data streams.

**Results:**

SEA scores represented a meaningful comparison of what participants encountered and what they engaged with. Operationalizing codes as either “present” or “absent” in a particular data stream allowed us to inspect not only which credibility cues participants engaged with with the most frequency, but also whether participants noticed the absence of cues. Code co-occurrence frequencies could thus indicate case-, time-, and context-sensitive information appraisal that also takes into account the quality of information encountered.

**Conclusions:**

Using SEA allowed us to retain epistemic access to idiosyncratic manifestations of both stimuli and engagement. In addition, by using the same coding scheme and designated co-occurrences across participants, we were able to pinpoint trends within our sample and subsamples. We believe our approach offers a powerful analysis encompassing the breadth and depth of data, both on par with each other in the feat of understanding organic, web-based search behavior.

## Introduction

### Background

In recent years, there has been a marked increase in online health information–seeking, with more than half of European Union citizens aged 16‐74 years reporting such behavior [1] and global trends exhibiting similar tendencies [2]. Web-based information-seeking merits particular attention, as the current information ecosystem is riddled with contradictions and inaccuracies [3]; many concerns have been raised about the quality of online health information encountered by users [4]. In this information ecosystem, users must undertake an active role in evaluating the trustworthiness and accuracy of content [4-6], but studies have shown users demonstrating poor skills in this area [7,8]. An improved understanding of how user characteristics and mental models influence web-based behavior is needed for interventions aimed at improving the retrieval of high-quality information.

Digital health literacy (DHL)—the ability to find, understand, and evaluate health information in digital environments and apply it to health behavior [9,10]—is a core competence for navigating online information and making decisions [2], which is associated with general health as well as several of its determinants [11]. Higher levels of DHL have been found to correlate with better health outcomes [4,12], desirable health behaviors (eg, prevention and management of chronic disease) [10], and a well-functioning patient-physician relationship [2]. Vulnerability to low DHL is most common among populations with higher age, lower socioeconomic position, underserved neighborhoods, and degraded physical environments [3].

DHL is commonly conceptualized as a formative construct consisting of four subconstructs: ability to (1) access or obtain (find), (2) understand, (3) appraise (evaluate), and (4) apply information relevant to health [3]. Past studies on DHL have primarily measured the respondent’s own assessment of their health literacy as applicable to digital contexts, which may aid in obtaining estimates of an individual’s confidence in their own skills. This self-assessment requires considerable metacognitive skills, yet often fails to provide valid measurements of the target construct [13]. Other studies have observed users using their DHL in online environments, but these have been limited to mock sites [14], confined search spaces [15-17], and outcome measures that do not yield much information on the drivers of behavior (eg, search time, queries, clickstream data [18]). Hence, such studies capitalize on a participant’s ability to retrieve specific information and the usability of a particular website, but do not lend thorough insight into organic searches, that is, nondirected browsing behavior during ill-structured tasks (ie, problems that are not clearly defined, which have multiple solution paths [19]).

Many scholars argue for the primacy of the subconstructs appraisal or evaluation within DHL. The evaluation of the quality of encountered content often focuses on the credibility or trustworthiness of information, which is understood as perspectival to the user and not an innate feature of information. Yet, credibility is commonly thought to be influenced by cues that surround the presentation of information [20]. Several studies aim to create lists of such credibility cues (relatively stable structural web page features, eg, contact information, links to external websites [21]) from participant narratives, usually gathered with concurrent and retrospective think-alouds (TAs) during organic or restricted search activities [8]. Other research has yielded credibility criteria checklists that aim to generalize credibility cues, such as HON scores, JAMA Benchmarks, the EQIP Tool, and the DISCERN Tool [22].

The existence of these two general approaches draws attention to the fact that consuming (obtaining, understanding, appraising, and using) good quality health-related information online depends crucially on (1) the quality of encountered information and (2) the user’s ability to recognize the quality of encountered information. Yet, few studies take into account both these aspects of information consumption simultaneously.

The objective of our feasibility study is to explore a method to bridge these two aspects of health-related information consumption and grant even consideration to both the stimulus (web page content) and the user (ability to appraise encountered content) within the context of web-based, organic search behavior. We examined novices and experts in information retrieval and appraisal to demonstrate a novel approach, stimulus-engagement alignment (SEA), which we situate as an extension of information foraging theory.

### Information Foraging Theory

Information foraging (IF) guides research on how users navigate among sources of online information, as well as predicts their behavior [23]. The theory stipulates that humans “forage” for information as other animals forage for food. An individual’s information environment consists of a combination of (1) stimuli (verbal, visual, etc) that are perceptually accessible (eg, text in a book, a diagram on a website) and (2) opportunities for interaction that increase perceivable information (eg, by scrolling down on a web page or clicking on a hyperlink). Information environments are “patchy” because information is clustered in certain areas, for example, on bookshelves, in libraries, or on websites [23,24]. Individuals generally engage with their information environment to achieve an active information goal, to obtain declarative knowledge about the world.

IF states that a user’s navigation in and among information patches depends on “information scent”; a high information scent leads to foraging behavior that aids the user in achieving an active information goal [23]. Information scent is conceptualized as a property of the stimulus [17]; users engage with environmental stimuli that are assumed to have “the maximum expected utility” in achieving an information goal [23], that is, certain content has higher information scent compared to others. Some authors using IF acknowledge the user perspective in determining information scent conceptually, such as Nwagwu stating that it is “the perceived value of a source of information based on cues such as links, abstracts, or summaries” [25], but the theoretical framework and computational models do not have a straightforward way of incorporating user appraisal.

In work thus far, chiefly by Pirolli et al [23,26], information scent has been computed with semantic similarity (proximity of words within documents in a corpus) with natural language processing techniques, such as latent semantic analysis and latent Dirichlet allocation. A chosen corpus and the derived word proximity scores form what is referred to as a “scent database,” which, in turn, serves as the basis of computing information scent. Typically, researchers use a large textual database (eg, a corpus containing thousands of news articles) and, since proximity scores are derived from associations between words in the task description and textual environmental stimuli, they are considered uniform across users who share an information goal. Associations between content and goal can exhibit different strengths: a strong association is interpreted as high relevance to the goal. Association strengths among words in human memory are assumed to be related to the probabilities of word occurrences and co-occurrences. Due to the fact that this understanding of information scent relies on word-based, standardized associations, it does not easily accommodate varying user representations of utility or meaning. This operationalization of information scent also fails to take into account the correspondence between encountered stimuli and their appraisal, as users in IF studies are generally not asked to evaluate or interpret the content they encounter.

### Proposed Modifications to IF Theory

#### Modifying the Concept of Information Scent

We define information scent as a person’s representation of the extent to which investing in a given patch of their information environment will aid progression toward a given information goal; thus, information scent is constituted by both the user and the stimuli in the information environment. Patches in themselves cannot have information scent: instead, they have attributes that contribute to information scent, but with varying degrees chiefly depending on the users’ extant internal representations. Hence, information scent can vary in the same person over time and context, depending on which information goals are active at any given time and the knowledge they have accumulated over a task. One implication of this definition is that, as opposed to previous studies (eg, [26]), different users can have different information scent values for the same patch; in addition, as a person progresses toward their information goal, the information scent of a given patch can increase or decrease.

#### Replacing Semantic Similarity With Code Co-occurrences Computed With SEA

We used code co-occurrences instead of semantic similarity to compute information scent. Codes are labels attached to data fragments that denote the observation that that fragment expresses a construct of interest (in our case, credibility cues). Owing to their conceptual nature, we assume that codes are more adequate at capturing meaning than individual words alone, and therefore they may be used to create more accurate models of the data. Furthermore, codes can be applied to nontextual credibility cues as well, hence opening up possibilities to move beyond the study of textual cues, which have characterized relevant research in this area thus far [25]. We define code co-occurrence as the state where two codes are applied within the same patch. Our scent database is constituted by the co-occurrence of particular, deductively applied codes within designated segments of data, and these code co-occurrences are computed with SEA. In this process, we compare the presence of code pairs in lines of data representing encountered online content and user engagement, and control for the number of visited patches to arrive at SEA values ranging from 0 to 1.

To examine the feasibility of using SEA in studying the proposed modifications to IF theory, we explore information scent (SEA values) for two groups (novices and experts of information retrieval and assessment) in their performance of organic, web-based information-seeking regarding a health-related subject.

## Methods

### Ethical Considerations

Ethics approval was gained from the University of Wisconsin-Madison Institutional Review Board. The study (2022‐0588) was determined to meet the criteria for exempt human subjects research as defined under 45 CFR 46:(3)(i)(B). Written informed consent to participate in the study and make their anonymized data public was obtained from participants before study participation. In accordance with open science principles, our entire project (eg, preregistration, recruitment materials, data collection instruments, codebook, analyses), including our data, is publicly accessible in our Open Science Framework repository [27]. Participants were compensated for their time with a US $30 Amazon gift card.

### Sampling Considerations

We sampled from two populations of internet users in our study. The first population consists of experts in information retrieval and assessment: individuals who have educational or work experience as librarians, journalists, or related professions (the expert subsample). The second population includes individuals with different backgrounds (the novice subsample). Our general sampling strategy was purposive, and we aimed for homogeneity within and across subsamples with respect to the following criteria (references support the criterion’s relevance in literature affecting computer skills and information literacy): geographical region [28] (limited to Wisconsin, United States), age [29] (chosen range: 18‐39 years), and since the search task involved an embedded biasing advantage in knowledge of Chinese language or culture, we aimed to exclude participants affected by these (ie, knowledge of Mandarin and Chinese ethnicity were exclusion criteria). We included 10 participants in both subsamples (N=20), which was (1) adequate to test the feasibility of our framework and tool and (2) feasible, as we used manual coding for all data types and had to plan realistically in terms of what we were capable of curating and coding within our time frame.

### Data Collection

The expert subsample was recruited via email from the University of Wisconsin-Madison Information School, as well as from the following departments: (1) Library and Information Studies and (2) Journalism. The novice subsample was recruited via a university mailing list. We used observational and TA protocols to collect data on information foraging within the framework of an interview. Our observational protocol contained the specifics of observing online foraging in a 10-minute search task with the information goal of learning about various COVID-19 origin theories; the task description is displayed in Textbox 1.

Textbox 1. **Description for online search task.**

"Some people think that the SARS-CoV-2 virus, which causes the disease COVID-19, originated in a laboratory in Wuhan, while others think that the first infection was caused by the virus being transmitted from a bat to a person. There are many theories on where and how the virus originated. Do some research on this topic online and, after 10 minutes, I will ask you some questions about the origin of the virus.”

The retrospective TA protocol standardized how self-reflection on behavior was elicited following the task, namely, questions to pose when the participant enters a web page, leaves a web page, or engages with content. Questions probed general impressions and indicators of trustworthiness for all visited web pages, as well as why participants engaged and disengaged with them. Both protocols were conducted online via a videoconferencing platform. Additionally, we used a pretask survey to collect basic sociodemographic data (detailed in the next section). Thus, the data collection process yielded survey data, as well as one video (10-minute task) and one audio-video recording (retrospective TA) per participant.

### Specifying Case and Patch Attributes

#### Case Attributes

Responses were downloaded from our survey platform in a CSV file; case attributes were specified in YAML, a human- and machine-readable data serialization language. The following variables constituted our case attributes: caseID, groupID (subsample), sex, gender, ethnicity, race, nationality, level of education, political affiliation (see the codebook in our repository for more on these variables). In this study, we used caseIDs and groupIDs for analytical purposes; other variables only constituted part of the sample description.

#### Patch Attributes

We defined patches as physical or virtual environments where produced and/or curated information can be stored and potentially accessed by users; we operationalized patches as any type of file that a browser can render when a user visits a particular URL. This included search engines, stand-alone PDFs, and pictures viewed on a separate page. Table 1 contains patch attributes and their descriptions, which were logged in the plain text files constituting the screen content data stream and represented in YAML, as shown in Textbox 2.

**Table 1.** Examples of patch attributes and how they were represented in YAML.

Attribute	Description	Examples
Patch tag	Nonunique label assigned to describe main website content and generator of content	PubMed, CNBC news
Domain	Second and top-level domain (and subdomain, where applicable)	google.com, ncbi.nlm.nih.gov
Patch identifier	Contains case identifier and the ordinal numbering of patches within a case’s search	patch_001_1, patch_002_15
Patch type	Categorical value indicating general type/function of website	Engine, SERP^a^, page, file
Start time	Start of activity on patch (marked by change in URL)	“00:02”
End time	End of activity on patch (marked by leaving patch, ie, closing the tab/window or moving to another one or change in URL)	“00:15”

a SERP: search engine results page.

Textbox 2. **Attributes in YAML.**

– – –

ROCK_attributes:

–

 patchTag: "UW-Madison Libraries"

 domain: "library.wisc.edu"

 pid: "patch_006_1"

 patchType: "engine/SERP"

 start: "00:00"

 end: "00:03"

–

 patchTag: "Google All"

 domain: "google.com"

 pid: "patch_006_2"

 patchType: "engine/SERP"

 start: "00:04"

 end: “00:08”

– – –

Patch tags were fully inductive, based on the URL and/or page title. Patch attributes “start” and “end,” indicating when a participant entered and left a patch, were transformed into the variable “totalTime” containing the number of seconds spent on a patch; for more on data transformations, see the “Data_Transformations” directory within our repository. For a full description of patch attributes, see our codebook (available at [30]).

Although technically an attribute of patches, the type of content they exhibited were specified as “states” to enable exploring transitions between patches (for more information, see the “Analyses and Modeling” section). Patch content codes were developed inductively by 2 coders independently reviewing the entire dataset, triangulating their tentative categories in one round, resolving differences via social moderation, and finalizing the 6 categories contained in Table 2.

**Table 2.** Codebook containing patch content type labels, definitions, and examples.

Code label	Definition	Examples
Academia	Websites of scientific institutes with academically approved content	Wiley, University of Illinois, National Library of Medicine, PubMed
Government	Websites of governmental organizations and other authorities, both national and international	Center for Disease Control and Prevention, US Department of Defense, World Health Organization
News	News sites with content often created by journalists targeting the general public	CNN, *The New York Times*, Al Jazeera
Science or health communication	Science- or medicine-related websites, with no academic affiliation	Wikipedia, WebMD, Nature
Other	Miscellaneous websites (eg, social media sites, nongovernmental organization websites)	Reddit, Zoom, Bat Conservation Trust
Engine/SERP^a^	Search engines and SERP	Google, Google Scholar, Illinois Library

a SERP: search engine results page.

### Data Preparation and Coding

#### Overview

Three multimodal data streams needed to be wrangled and synchronized: human-computer interactions (HCI) and screen content from the task completion video, as well as narratives from the TA video. We aimed to represent coded data in a single, tabularized dataset for further processing. Our iterative code development process is documented in detail in our repository under “Operationalization\Code_development.”

#### Data Stream: Think-Aloud

Audio data from the TAs were transcribed in an automated process, manually corrected for accuracy, anonymized, and placed into separate plain text files per data provider. Using the R package {rock} [31], we segmented the data [32] by sentence (a newline character was added after punctuation marks) and assigned each one a unique “utterance identifier.” Coding was performed on this level of segmentation. Codes capturing (textual and nontextual) credibility cues were developed inductively from the entire dataset under the parent code Appraisal (APPR) and included patch features that contributed to participants’ assessment of content and creator. Note that in this study we only disclose codes that were used for SEA; for our full coding scheme, see [33]. Our coding process involved several phases:

1. Free inductive coding performed autonomously by 2 raters.
2. Triangulation and creation of a tentative codebook.
3. Test coding performed autonomously by 2 raters on the same subset of data.
4. Triangulation and repetition of steps 2 and 3 for several iterations until a final codebook was developed.
5. Interrater reliability testing (using Cohen κ [34] as indicator) to confirm shared understanding and pinpoint discrepancies.
6. Triangulation and repetition of step 5 (until Cohen κ ≥0.90 was reached for all codes).
7. Deductive application of final coding scheme to full dataset.

Relying on the final codebook, one researcher coded the corpus manually with the Interface for the Reproducible Open Coding Kit (iROCK, available at [35]). To ensure consistent application of codes, we computed intrarater agreement with Cohen κ when 50% of the dataset was coded, and when the last interview was finished, both instances yielded a κ of at least .70 per code. Table 3 displays a simplified version of TA codes within our codebook.

**Table 3.** Codebook containing code labels, definitions, and examples for codes describing credibility cues (APPR).

Code label	Definition	Examples
Date	Date content was created, modified, or updated	So it’s from 2022.
Author	Author(s) names (full names or abbreviations, acronyms, pseudonyms)	It had multiple researchers on it too. So, like more eyes on, it could give it more credibility.
Author affiliation	Author affiliation, organization (via textual or visual designation, eg, seal or logo)	They’ve put their seal on it [...] and they’re willing to put it to their name, they’re willing to defend it.
External references	Hyperlinks, citations, quotes with references, footnotes, bibliography	That’s just a warning sign to me as, as trustworthy, like, this is a fact not cited.
DOI	Digital object identifier	There’s the DOI.
Trusted URL	High-level domains, secure servers or padlock; implemented encryption	But I will admit that it looked like a more professional website than the CNN one in the sense that it had the .org.
Scientometrics	H-index, number of citations, Scimago quartile, impact factor	I was actually looking for the impact factor, doesn’t tell me an impact factor.
Advertisements	Structural elements with commercial intent separate from the main content	It has a clean, ad-free homepage.

Each of the 8 codes manifested as either a “presence” or an “absence” (eg, Date_pres or Date_abs), as both their presence and their absence was of interest in all 3 data streams, resulting in a total of 16 codes. The identifiers of all codes used in the TA data stream were prepended with the slug “TA_.”

#### Data Stream: HCI

HCI observations were transcribed in a specific template as follows: “Action | Content | Location.” In this template, Action referred to physical engagement with the computer: type, click, scroll, hover, and highlight. Content indicated what the action referred to, what it was performed on, or a specification of the action (eg, the object selected, the characters typed in, or the direction of a scroll), while Location signified the place of action within the window (eg, search bar, page, hyperlink). For more details on this template and its code development, see [33]. Transcribed HCI data were placed into separate plain text files per participant; codes were adopted from those developed for the TA data stream, their identifiers were prepended with the slug “HCI_” and applied with iROCK. HCI codes did not include a “present” versus an “absent” version, as the “absence of HCI” (eg, a user *not* interacting with a date) was not considered an element of HCI. Thus, HCI codes were constituted by those listed in Table 3.

#### Data Stream: Screen Content

Screen content, that is, the content of web pages visited by the participant over the 10-minute search, was documented as a list of patch identifiers (pids). These identifiers were unique across cases (ie, no repetition of pids across participants), but nonunique within cases (ie, a participant could revisit a previous patch). The pids constituted the data in this data stream and were placed in separate plain text files per participant. Codes were adopted from those developed for the TA data stream; their identifiers were prepended with the slug “SC_” and applied with iROCK.

#### Aligning Data Streams

**Figure 1.**
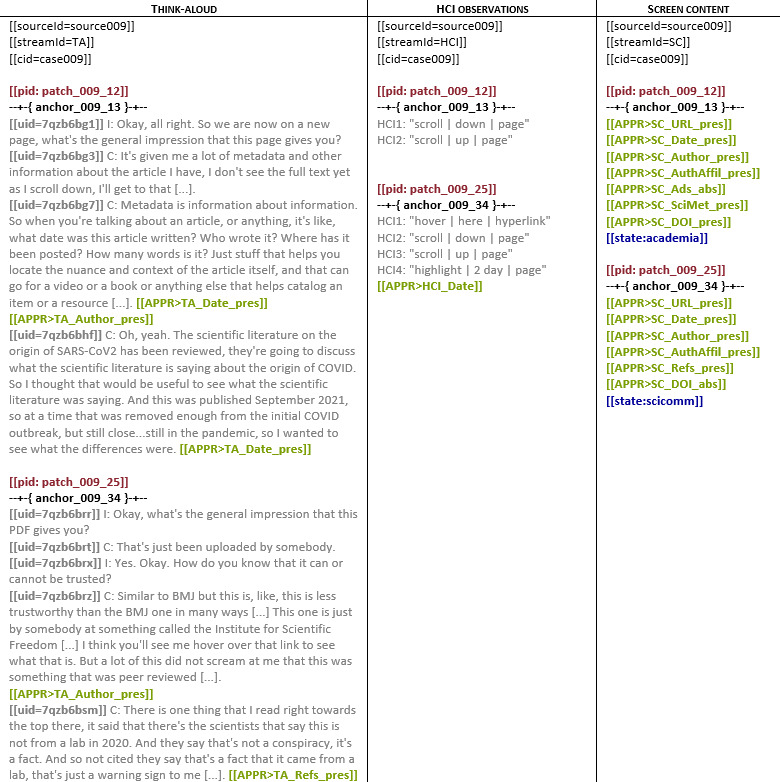
Three data streams with data (gray), source/stream/case identifiers (black), patch identifiers (red), utterance identifiers (gray bold), code identifiers (green), and state identifiers (blue), as well as anchors (black bold). HCI: human-computer interactions.

To compare engagement (HCI; what participants did on a web page) and mental models (TAs; what participants said regarding a web page) with patch features (screen content), we aligned the 3 data streams by embedding anchors (text strings) after each patch in every source. We used the {rock} functionality “Anchor-based Stream Synchronization” to map codes from all streams onto a primary stream (TA data). Figure 1 illustrates 3 streams of data for 2 patches, with source, stream, case, patch, utterance, code, and state identifiers, as well as anchors specified according to the Reproducible Open Coding Kit standard.

### Tabularization and Transformation

We parsed the coded and aligned data streams with the {rock} package, creating an R object that contained all data from all participants in tabular form, as well as case and patch attributes. Rows in this dataset were constituted by data segmented and aligned according to anchors (delimiting patches); columns contained data from the 3 data streams, codes (in binary form: 1 if a code was present, 0 if it was absent in the line), state identifiers, and case identifiers, as well as case and patch attributes.

To prepare for SEA computation (comparing code co-occurrences in the screen content data stream versus the other two streams), we created a new variable that combined the codes in the TA and HCI streams. If a code was present in either or both of these, the derived variable (TA-HCI) value was 1; if it was absent in both, then the value was 0. This procedure was performed separately for all codes per patch. Subsequently, we generated SEA scores per patch by comparing the TA-HCI values with those in the screen content stream. Table 4 contains the code “date present” in 3 data streams for 5 patches (the HCI code has no present/absent versioning), as well as the derived variable TA-HCI and the SEA score.

**Table 4.** Code “date present” manifesting in 5 patches and 3 data streams, with the derived variable TA-HCI (TA-HCI_Date_pres column) and SEA value (SEA_Date_pres column), as well as a qualitative description of all possible co-occurrences (final column).^a^^,^^b^

Patch	TA_Date_pres	HCI_Date	TA-HCI_Date_pres	SC_Date_pres^c^	SEA_Date_pres	Qualitative description
1	1	0	1	1	1	Credibility cue was present in stimulus and the participant engaged with it (in think-aloud)
2	0	1	1	1	1	Credibility cue was present in stimulus and the participant engaged with it (in HCI)
3	0	0	0	1	0	Credibility cue was present in stimulus but the participant did not engage with it
4	1	0	1	0	0	Participant engaged with a credibility cue (in think-aloud) that was not present in the stimulus
5	0	0	0	0	0	Credibility cue was not present in the stimulus and the participant did not engage with it

a SEA: stimulus-engagement alignment.

b TA-HCI: think-aloud human-computer interactions.

c SC: screen content.

Our scent database consisted of relevant co-occurrences for all 16 codes (present and absent version of the eight codes disclosed in Table 3) applied to the 3 data streams. Thus, for our sample, we obtained a total of 16 SEA values, per visited patch, for each participant’s 10-minute search.

### Analyses and Modeling

#### Descriptive Analyses

To illustrate the presence and absence of credibility cues in the patches visited by experts and novices, we created histograms indicating the frequency of codes in the screen content data stream of each participant (16 codes total, maximum 1 occurrence per patch). We also computed the likelihood of a participant transitioning from one patch content type to another (designated as “states”; see Figure 1) with Qualitative/Unified Exploration of State Transitions (QUEST). As part of {rock} functionality, QUEST generated Markovian models of transitions between patch content types based on a state transition network where frequencies of transitions from a state to itself and other states constituted the total transition counts for each state. QUEST models were produced from our tabularized dataset with transition counts and probabilities per participant, rounded to 2 decimals.

#### SEA Analysis

SEA scores only pertained patches that were not search engines or search engine results pages (SERP), as these patches exhibited incomparably different credibility cues than other, substantive web pages. SEA scores were generated by computing the proportion of 1s in each column (for each of the 16 codes) per participant, thereby controlling for the varying numbers of patch visits during the 10-minute search.

## Results

### Sample Characteristics

**Figure 2.**
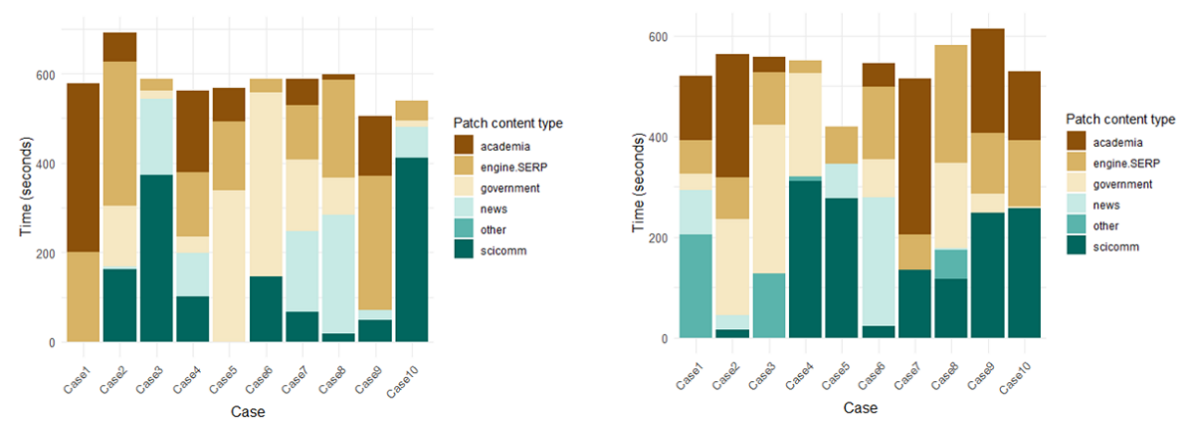
Time spent on patch content types per case for both subsamples. SERP: search engine results pages.

Our sample consisted of experts (n=10; 6 males and 4 females) and novices (n=10; 5 males and 5 females) of information retrieval and appraisal. Nine of 10 experts and 2 of 10 novices had earned at least a bachelor’s degree; all participants had completed secondary education. The expert group included participants originally from the United States (n=8), Nigeria (n=1), and Pakistan (n=1), whereas the novice group consisted of participants from the United States (n=4), the United Kingdom (n=2), France (n=1), Brazil (n=1), and Russia (n=1). All participants were residing in Madison, Wisconsin, at the time of data collection. Participants visited a total of 439 patches (patch types: page n=205, SERP n=152, engine n=64, file n=18) with 306 unique patch identifiers. On average, participants in the expert subsample visited 20.4 (SD 11.76) patches per 10-minute search, while novices visited 16.9 (SD 7.01). Figure 2 displays the distribution of patch content types within the 10-minute participant searches.

Experts spent most of their 10-minute search on search engines and results pages, while novices spent most of their time on patches characterized as scicomm (duration in seconds, for experts and novices, respectively: engine/SERP=1566 vs 1054; academia=906 vs 1104; government=1199 vs 1011; news=812 vs 444; scicomm=1326 vs 1385; other=0 vs 398).

### Qualitative/Unified Exploration of State Transitions

**Figure 3.**
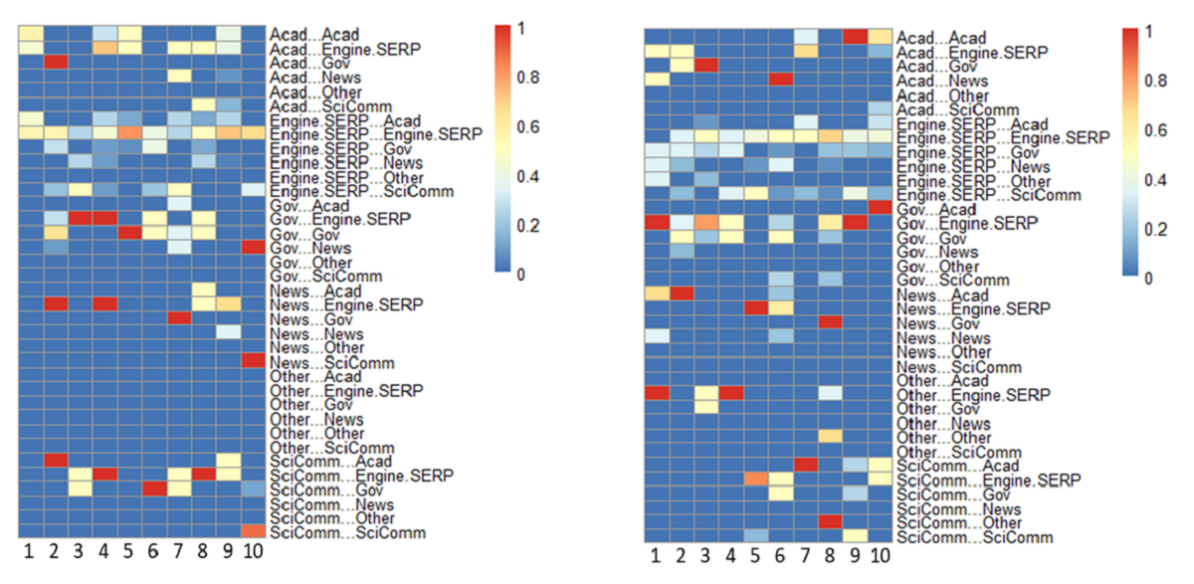
QUEST transition probabilities for patch content types per case for both subsamples. The codebook is provided in Table 3. QUEST: Qualitative/Unified Exploration of State Transitions; SERP: search engine results pages.

Figure 3 displays the transition probabilities between patch content types (for definitions, see Table 2). When a particular patch type and/or transition did not appear in a source (not applicable), they were designated a value zero in the heat map.

Of all possible transitions, participants in the expert subsample were most likely to transition from engine/SERP patches to those in the same category (self-loop), while novices were most likely to transition from patches labeled government or other, back to a search engine or results page. Experts usually selected to visit academic or government-curated patches from SERPs, while novices preferred government or scicomm patches. The highest transition probabilities among experts were from government, academic, and news sites back to search engines or results pages. Patches labeled other were only observed in the novice group (eg, Council on Foreign Relations, The New Reddit Journal of Science). Of applicable transitions, the expert subsample was least likely to transition from government patches to academic ones and to exhibit self-loops among news sites. Novices were least likely to transition from government patches to scicomm ones and to select patches labeled other from their search results.

### SEA Analysis

**Figure 4.**
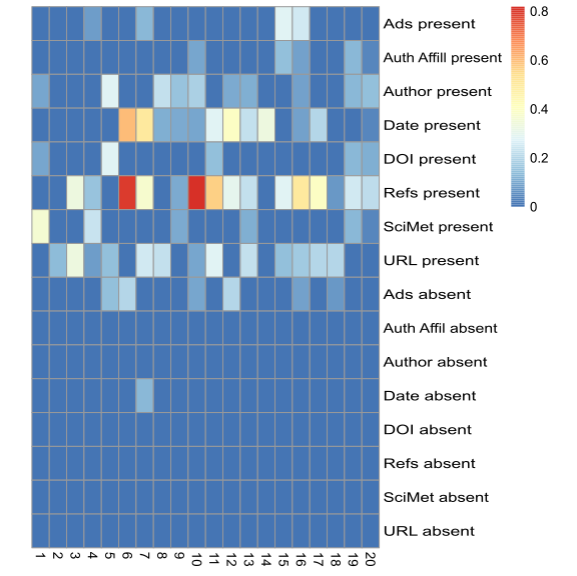
SEA results showing the alignment of 16 codes (presence and absence of credibility cues) in the stimulus data stream with those in either human-computer interactions or think-aloud data for experts (1-10) and novices (11-20). The codebook is provided in Table 3. SEA: stimulus-engagement alignment.

To understand how encountered content and its appraisal manifested regarding credibility cues, we used SEA analysis to compute alignment between content and its perceived credibility. Figure 4 shows the alignment of stimulus and participant engagement codes (labels are capitalized) in our 3 data streams per patch, aggregated over the 10-minute search participants performed. In both subsamples, the strongest alignment occurred for external references present (experts n=6, ∑=2.58; novices n=9, ∑=2.81) and date present (experts n=5, ∑=1.40; novices n=7, ∑=1.56). The absence of credibility cues was, in general, disregarded by participants, with the exception of the absence of advertisements; one participant (expert 7) noted the absence of date multiple times as an indicator of questionable trustworthiness.

Although the 2 subsamples averaged similar SEA scores for all codes (0.05), experts engaged with a total of 35 credibility cues, while novices engaged with 42. Experts tended to engage with fewer cues but with higher frequency, which may suggest that expertise in information retrieval and appraisal limits the focus of attention to certain cues (eg, external references, date, and trusted URL) at the expense of others.

### Comprehensive Comparison

**Figure 5.**
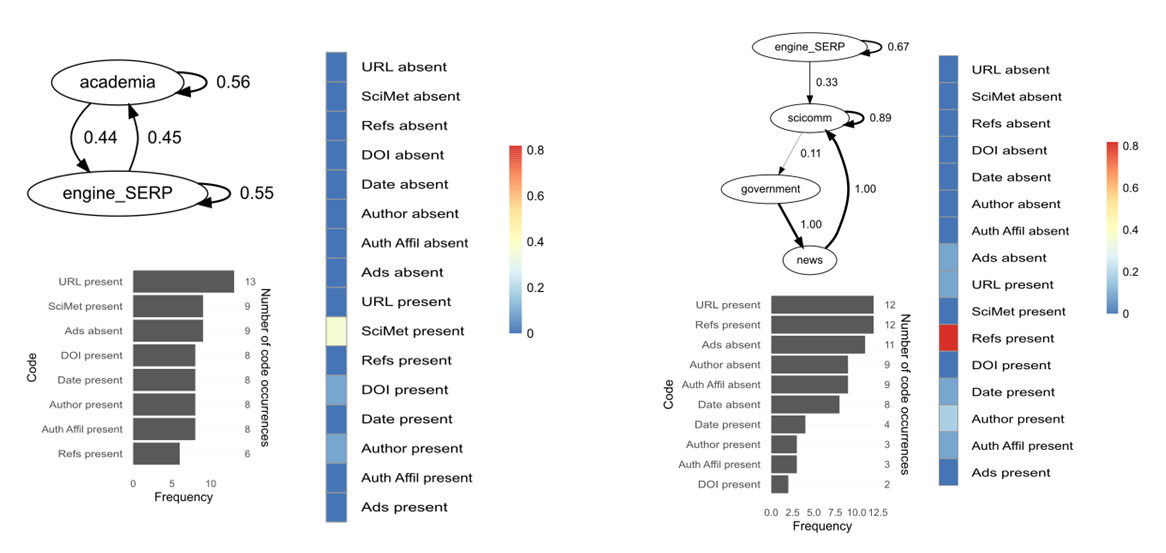
Comparison of 2 experts based on QUEST (top left), code frequency histograms (bottom left), and SEA scores (right). The codebook is provided in Table 3. QUEST: Qualitative/Unified Exploration of State Transitions; SEA: stimulus-engagement alignment.

In this section, we use QUEST (transition probabilities for patch content types), SEA (designated code co-occurrences between encountered credibility cues and engagement with them), code frequency histograms (from coded screen content data), and qualitative insight (via hermeneutics) to compare 4 cases. Cases 1 (female) and 10 (male) were in the expert subsample, while cases 13 (female) and 14 (male) were in the novice subsample. Figures5  6 show the QUEST models, SEA scores, and screen content code frequency histograms for 2 experts and 2 novices, respectively.

**Figure 6.**
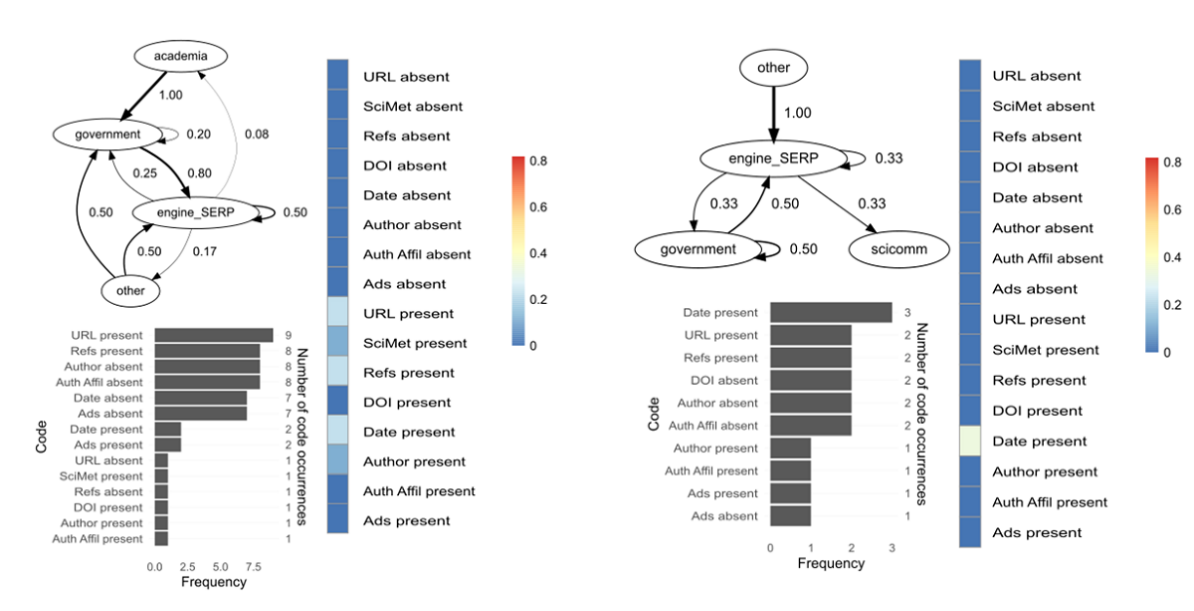
Comparison of 2 novices based on QUEST (top left), code frequency histograms (bottom left), and SEA scores (right). The codebook is provided in Table 3. QUEST: Qualitative/Unified Exploration of State Transitions; SEA: stimulus-engagement alignment.

Expert 1 conducted a well-defined search with logical operators (eg, “(‘COVID-19’ OR ‘novel coronavirus’ AND ‘Wuhan’ OR ‘Origin’ AND (‘bat’))”) and based retrieval on scientometric considerations, such as: “I would go to SJR to check the journal ranking [...] this journal has a long history of being in Q1.” Congruent with their focus in retrieval, the participant appraised the trustworthiness of patches primarily based on scientometrics, while displaying limited engagement with other cues (eg, external references, author affiliation, date). The most common credibility cue present in the encountered stimuli was trusted URL, followed by scientometrics and the absence of advertisements. Expert 1 spent most of her time on academic patches and exhibited 20 state transitions among 14 unique web pages; she solely visited academic patches following searches (ie, engines were used to retrieve academic patches only) and was most likely to transition from patches with academic content back to that same patch content type. She was just as likely to use a search engine to find academic patches as to be prompted to search for information that she encountered on patches labeled academia. Thus, expert 1’s retrieval and appraisal were all centered around scientometric indicators, and she exhibited an equal likelihood in transitioning from and to search engines and results pages.

In contrast, expert 10 did not visit any academic patches and was most likely to select scicomm patches from the search results page, which is the patch type he spent most of his time on. Expert 10 exhibited higher SEA scores for more credibility cues (n=6) compared to expert 1 (n=3), of which external references retained the highest frequency. The participant emphasized the primacy of references or citations in the text body during information appraisal: “At this point, I was looking at the quotes that they were citing [...] So again, with Wikipedia, trusting the editorial process there, and also the multitude of hyperlinks to other articles that have also gone through that editorial process.” Congruently, expert 10 visited a high number of patches exhibiting external references, but cues such as author, author affiliation, and date were more often absent than present in the stimuli. He exhibited 14 transitions among 13 unique web pages, with a strong self-loop in scicomm; he was likely to transition from this category to patches labeled government. From government-curated information, he was likely to transition to news sites and back to those labeled scicomm. Expert 10’s QUEST diagram indicates that no searches were performed for new information on encountered patches. In general, expert 10 engaged with the presence of several credibility cues, but frequently encountered patches lacking in these cues (eg, DOI, author, author affiliation) and did not engage with those absences.

Novice 13 spent most of her search on government patches and engaged with 5 credibility cues during her 10-minute search, such as author affiliation and trusted URL. Nevertheless, she also expressed uncertainty in how these could apply to trustworthiness: “Um, honestly, there’s no real, real good way to know whether this could or couldn’t be trusted.” Credibility cues such as author, author affiliation, and date were often absent in the stimuli, but the participant did not engage with these absences (which would have resulted in higher SEA scores). Of their 20 transitions total (12 unique web pages), novice 13 was most likely to transition from academic patches to government ones. After performing a search, she was most likely to click on a patch labeled government and those visits often ended with the participant searching for information she had found on the government site; this was true for patches labeled other as well.

Novice 14 spent most of his search on scicomm patches. He did not engage with any credibility cues apart from date, which meant disengaging from other cues, such as external references and trusted URL, even though most visited patches exhibited these. Many visited patches lacked DOI, author, and author affiliation, but the participant did not engage with these absences. Novice 14 exhibited 6 transitions, all unique web pages, and was most likely to visit government and scicomm patches following a search. Government self-loops exhibited a high probability, which could indicate a trust in government-generated information, but the participant’s think-aloud data demonstrates otherwise: “It’s basically lying with statistics, you can always find somebody that will make the statistics say what you want. [...] You have to look at reality, reality itself, like, people used to think leaded gasoline was fine. And that’s what the majority of scientists said. Scientists and doctors used to say that smoking was healthy for your child while you were pregnant.” Thus, novice 14 visited relatively few patches exhibiting a fair number of credibility cues, but did not engage with those cues nor the absence of other cues. In addition, despite viewing mainly government-curated information, the participant expressed distrust concerning that information.

### Notes on Feasibility

We explored the possibility of granting even consideration to stimulus and user in modeling information consumption. The co-occurrence of designated codes in the stimulus versus other 2 data streams represented a meaningful comparison of what the participant encountered and what they engaged with. Operationalizing codes as either “present” or “absent” in a particular data stream allowed us to inspect not only which credibility cues participants engaged with the most frequency, but also whether participants noticed the absence of cues. This, in turn, served to differentiate stimuli exhibiting a high or low number of cues (measured via their presence and absence), reflective of the trustworthiness of their content. Code co-occurrence frequencies could thus indicate tailored information scent, which also takes into account the quality of information encountered. Retaining individual SEA scores (see Figure 4) allowed us to pinpoint participant idiosyncrasies concerning which credibility cues participants most frequently engaged with and whether the absence of those cues was cognized. Refraining from aggregation also enabled a more complex within-case exploration using different analyses to show the following:

1. The alignment between that which was encountered and engaged with (SEA analysis).
2. The quality of encountered stimuli (code frequencies).
3. The probability of transitioning between certain types of content (QUEST).
4. Qualitative insights into behaviors and mental models (hermeneutics).

SEA scores were the product of several methodological decisions, most notably code development (eg, from which data stream or streams codes were generated and in what way were their definitions coordinated across streams), code application (eg, choices as to whether instances of codes apply in the case of particular stimuli or acts of engagement), segmentation and alignment of data (eg, operationalizing a meaningful segment of data applicable across data streams), co-occurrence designation (ie, constructing a scent database from meaningful code co-occurrences), and co-occurrence computation (eg, mode of accumulation, weighting).

## Discussion

### Principal Findings

We tested a novel approach to studying information scent based on synchronized data from 3 qualitative data streams, comparing stimuli and user engagement via the novel SEA analysis. Using SEA allowed us to retain epistemic access to idiosyncratic manifestations of both stimuli and engagement and, through using the same coding scheme and scent database across participants, also allowed us to pinpoint trends within our sample and subsamples. Our comprehensive analysis involving SEA scores, code frequencies, QUEST, and hermeneutics afforded insights into how well credibility cues and their absences in stimuli aligned with participant engagement, what quality and types of content participants encountered, as well as their mental model of the stimuli and their own behavior. This exploration went further than merely indexing what web pages users encounter, or what their subjective insights on content trustworthiness may be, and offered a valid alternative to information scent that is case-, time-, and context-sensitive. We will now proceed to discuss implications intersecting this new method and IF theory.

### Bridging Stimulus and User

As a foundational premise of information theory, information is contingent on a sender and a receiver [36], which we presently translated to stimulus (features of online content) and user (mental model estimated via engagement manifesting in narratives and HCI). Information scent has been defined according to the “maximum expected utility” of a patch in light of an information goal, referring “to the local cues that users process in making such judgments” [26]. Although the information goal has a conceptual and operational role in IF-based models, such as SNIF-ACT (Scent-based Navigation and Information Foraging in the ACT architecture), the ability of a user to detect cues as well as their mental models of both the information goal and (features of the) patch are generally de-emphasized or lacking. To account for both sender and receiver, we proposed modifying the concept of information scent to include both stimulus and user, which allowed us to examine not only the presence or absence of credibility cues in visited content, but also whether or not the participant engaged with those. This innovation offers a feasible way to integrate user perception into IF models by creating a conceptual and computational role for both patch features and user mental models; more generally, it offers studies on DHL a way to compare the quality of online content with the user’s ability to recognize credibility cues within that content.

### Curating the Scent Database

Traditionally, a scent database is constructed from semantic similarity within a large textual corpus, constraining researchers to token-based analyses (eg, [37]). Using a corpus that is not tailored to the subject under scrutiny may entail relying on co-occurrences that are not meaningful (eg, merely lend information about the distribution of word forms) or are misleading in certain analyses (eg, represent and reproduce biases resulting from data, algorithms, and content presentation) [38]. Depending on analytical objectives, codes that represent higher-level constructs (eg, concepts and symbols that require longer sequences to represent) may be more adequate in capturing both textual and nontextual properties of the data [32]. We demonstrated how a scent database can be constituted by designated co-occurrences of codes in the data (stimuli and user engagement) that represent constructs of interest (credibility cues). These codes can be created inductively from the collected data, adopted from an existing coding scheme, or a combination of both. In our case, the inductively created codes showed almost complete overlap with those found in other studies [8], except for our code DOI. Designated code co-occurrences may provide a valid means of computing information scent, as they can denote a more meaningful association than the co-occurrence of words.

### The Primacy of the Information Goal

Most IF-based studies that define information scent as a property of the stimulus focus on the user’s ability to seek out content with high information scent (as per that definition) [25], as information goals typically include locating specific pieces of information with high utility in confined search spaces (cf [23]). Problem-solving literature often distinguishes between well- and ill-structured problems; while the former entails working with a finite number of concepts and rules in a constrained space with a well-defined initial and goal state, the latter connotes the integration of several content domains to reach nonpredictable or convergent solutions [19]. An example of ill-structured problem-solving is sensemaking, which is the act of seeking, integrating, and interpreting information to support decision-making [39]. When researching health-related information, sensemaking is often used to perform an organic search and process a variety of information, including untrusted or poor quality content. In these cases, finding the shortest path to good quality information is not as crucial as being able to correctly appraise information quality and integrate encountered content. For this reason, the stimulus-centric definition of information scent cannot be applied to models of sensemaking in a straightforward way, while SEA can potentially accommodate ill-structured problem-solving in nonrestricted search activities.

### Considering Discernment

With all primates, including humans, foraging for food is considered learned behavior [40]. Analogous to food foraging decisions, such as discerning edible from nonedible or estimating nutritional value versus nutritional requirements, we assumed that the recognition of both the absence and presence of credibility cues together constitute appropriate appraisal of information trustworthiness. In this conceptualization, information scent can still be high, despite the participant visiting an untrusted patch; similarly, the same patch can retain different information scent values for various participants or even differ for the same user over time. We believe this definition of information scent accounts for and more accurately represents the diversity of information environments and user engagement. More specifically, during ill-structured problem-solving, participants may visit patches with varying numbers of credibility cues to triangulate previously encountered information. A web page with few credibility cues may indicate less trustworthy information, but if the user is aware of this caveat, information derived from these patches may still be valuable in sensemaking processes. SEA analysis, used together with qualitative insight, can provide a more accurate interpretation of why a user visited certain types of (trusted or untrusted) content and how they integrated information into their mental model. Using mixed methods to create a rich dataset containing both user actions and perceptions [41] also sheds more light on the DHL dimensions of appraisal and understanding via being privy to participant idiosyncrasies in reasons for and implications of visiting patches with varying numbers of credibility cues. As our comprehensive analysis (QUEST, SEA, code frequencies, hermeneutics) showed, this allowed for complex within-case exploration.

### Alternatives in SEA Computation and Aggregation

Accounting for both stimulus and user, curating a scent database from meaningful co-occurrences, and aligning the concept of information scent to the (type of) information goal still leaves much methodological leeway in information scent computation. Although we “dummy coded” credibility cues in stimulus and engagement data, which served as the basis for SEA scores, weighted values could allow for a more refined representation of the presence and absence of credibility cues in stimuli and also for distinguishing between more or less vital cues in user engagement (eg, assigning more weight to author affiliation being present versus a DOI). Naturally, this would also entail justifying the rationale behind weighting choices. These methodological decisions depend crucially on study objectives and underlying epistemic assumptions, as does the aggregation of data across participants. In this study, SEA scores were summed and averaged per subsample to provide descriptive information and pinpoint general tendencies within subsamples.

### Limitations

Due to the Markovian model that was used, QUEST diagrams were only able to visualize pairwise transitions between patch content types, which implies that the full sequence of such patch types throughout the 10-minute search was not represented in the model. This connotes an understanding limited to the sequence and interaction of 2 patch types, rather than a complete string, which would have yielded unwieldy sequences hindering the identification of patterns within subsamples. The coding of the stimulus data stream, signifying a “gold standard” against which user engagement was contrasted, constituted a challenge, as not all codes were applicable to every patch. For example, while the date of publication can be expected to be present on all web pages with substantive content, a DOI may not be applicable to news reports or interviews. Further complicating this issue, some news sites provide a DOI to all published pieces, hence a DOI could be expected for those patches, but not necessarily for other news websites. These judgments of code applicability were made on a patch-by-patch basis by the coder responsible for coding the stimulus data stream; without a more systematic approach to determining code applicability, this connotes a threat to reliability. Furthermore, as with all such methodological decisions, the operationalization of patch and code co-occurrences constrained our analysis. Patches were considered unique across but not within search tasks, therefore it was not possible to (1) identify which content was accessed by more than one participant and (2) how information scent changed over the duration of a search. This information superseded the goals of this paper but could be obtained from the same data if coded differently. Additionally, although think-aloud and HCI data served as a valuable indicator of participants’ mental models of the information goal and content appraisal, engagement could also have been measured with other modalities, such as eye-tracking, which could provide further insight into user mental models.

Analytical gaze can and should be directed toward varying participant interpretations of the same cues. For example, even though 2 cases engage with scientometric indicators on a patch, the presence or absence of scientometrics, and the notion itself, may carry markedly different meaning for the 2 users. Additionally, as with any analysis based on qualitative coding, SEA scores are determined by coding and segmentation choices. These choices and their methodological implications are listed in the section “Notes on Feasibility” and have a profound implication for the interpretation of SEA results. Furthermore, as flattened data, SEA scores in themselves may be misleading (as mentioned in the “Concerning Discernment” section) and require qualitative insight to formulate, validate, influence, or question their meaning. Finally, we would like to reiterate that this was a feasibility study; hence, it is likely that our substantive results reflect sampling or other error sources rather than regularities in how people process health information. As such, although our conclusions concerning feasibility are solid, these substantive findings should be considered tenuous.

### Conclusions

Developing and deploying SEA as a means for information scent computation enabled us to compare patch features with user engagement and to analyze user-specific information scent values. Aside from aggregating data across cases to detect subsample-specific tendencies, via using the combination of anchor-based stream synchronization, SEA, QUEST, code frequencies, and qualitative insight, we were able to obtain a refined within-case perspective as well. We believe these tools lend a powerful analysis encompassing the breadth and depth of data, both on par with each other in the feat of understanding web-based search behavior. Future studies could benefit from exploring weighting in SEA variables, enabling a refined ranking of credibility cues (reflecting real-world phenomena under scrutiny) and designated co-occurrences of more than 2 codes (enabling more complex associations among variables). SEA analyses can be extended beyond credibility cues to any construct of interest (eg, query specifications or the comparison of understanding and appraisal versus implementation), supporting the exploration of other DHL dimensions.

## Abbreviations

DHL: digital health literacy
HCI: human-computer interactions
IF: information foraging
iROCK: Interface for the Reproducible Open Coding Kit
pids: patch identifiers
QUEST: Qualitative/Unified Exploration of State Transitions
SEA: stimulus-engagement alignment
SERP: search engine results pages
SNIF-ACT: Scent-based Navigation and Information Foraging in the ACT architecture
TA: think-aloud
TA-HCI: think-aloud human-computer interactions
